# Quality of the Development of Traumatic Brain Injury Clinical Practice Guidelines: A Systematic Review

**DOI:** 10.1371/journal.pone.0161554

**Published:** 2016-09-01

**Authors:** Anjni Patel, Mateus Mazorra Coelho Vieira, John Abraham, Nick Reid, Tu Tran, Kevin Tomecsek, João Ricardo N. Vissoci, Stephanie Eucker, Charles J. Gerardo, Catherine A. Staton

**Affiliations:** 1 Division of Emergency Medicine, Department of Surgery, Duke University Medical Center, Durham, North Carolina, United States of America; 2 Centro Universitário Unicesumar, Maringá, Brazil; 3 Duke Global Health Institute, Duke University, Durham, North Carolina, United States of America; 4 Division of Global Neuroscience and Neurosurgery, Department of Neurosurgery, Duke University Medical Center, Durham, North Carolina, United States of America; 5 Faculdade Inga, Medicine Department, Maringá, 87005240, Brazil; Fraunhofer Research Institution of Marine Biotechnology, GERMANY

## Abstract

Traumatic brain injury (TBI) is a leading cause of death worldwide and is increasing exponentially particularly in low and middle income countries (LMIC). To inform the development of a standard Clinical Practice Guideline (CPG) for the acute management of TBI that can be implemented specifically for limited resource settings, we conducted a systematic review to identify and assess the quality of all currently available CPGs on acute TBI using the AGREE II instrument. In accordance with PRISMA guidelines, from April 2013 to December 2015 we searched MEDLINE, EMBASE, Google Scholar and the Duke University Medical Center Library Guidelines for peer-reviewed published Clinical Practice Guidelines on the acute management of TBI (less than 24 hours), for any level of traumatic brain injury in both high and low income settings. A comprehensive reference and citation analysis was performed. CPGs found were assessed using the AGREE II instrument by five independent reviewers and scores were aggregated and reported in percentage of total possible score. An initial 2742 articles were evaluated with an additional 98 articles from the citation and reference analysis, yielding 273 full texts examined. A total of 24 final CPGs were included, of which 23 were from high income countries (HIC) and 1 from LMIC. Based on the AGREE II instrument, the best score on overall assessment was 100.0 for the CPG from the National Institute for Health and Clinical Excellence (NIHCE, 2007), followed by the New Zealand Guidelines Group (NZ, 2006) and the National Clinical Guideline (SIGN, 2009) both with a score of 96.7. The CPG from a LMIC had lower scores than CPGs from higher income settings. Our study identified and evaluated 24 CPGs with the highest scores in clarity and presentation, scope and purpose, and rigor of development. Most of these CPGs were developed in HICs, with limited applicability or utility for resource limited settings. Stakeholder involvement, Applicability, and Editorial independence remain weak and insufficiently described specifically with piloting, addressing potential costs and implementation barriers, and auditing for quality improvement.

## Introduction

Traumatic brain injury (TBI) is one of the leading causes of death and disability in both developing and developed countries, with the highest incidences among young people less than 30 years of age [1, 2]. While the current global burden is unknown, previous conservative estimates indicate an annual incidence of over 10 million people sustaining a TBI leading to hospitalization or death, with road traffic injuries causing a preponderance of cases [1, 3]. TBI incidence is projected to continue to rise worldwide due to the continued increasing rates of road traffic injuries, particularly in low and middle-income countries (LMIC) where the rates are twice as high as in high-income countries [1, 4]. Furthermore, the World Health Organization suggests that upwards of 90% of road traffic injury deaths occur in LMIC. These trends have been attributed to the rapid economic growth, urbanization, and motorization but limited infrastructure improvements in LMIC [1, 4].

Unfortunately, as the burden of TBI continues to increase globally, appropriate prevention efforts have been limited, especially in LMIC, and healthcare quality remains poor, resulting in disproportionately higher mortality rates [5]. One healthcare quality improvement measure that has shown impact is the use of clinical practice guidelines (CPGs) [6–9]. In fact, in the last several years a significant number of CPGs for acute TBI care has been developed worldwide [5]. However, CPGs vary in quality and comprehensiveness, leading to difficulties with standardization of care, adaptation and implementation, particularly in resource limited settings [10]. There is limited literature comparing and evaluating the strengths and weaknesses of all available CPGs for the treatment of acute TBI.

The Brain Trauma Foundation first developed CPGs for the management of severe TBI in the United States in 1995, with subsequent updated editions published in 2000 and 2007 [11–13]. These guidelines have gradually gained acceptance and increased use internationally, with a number of countries adapting them to their individual needs. For example, until recently in Saudi Arabia, patients with severe TBI were managed per individual provider knowledge and experience. By implementing an ICU protocol derived from the Brain Trauma Foundation’s guidelines, Saudi Arabian providers were able to significantly reduce hospital and ICU mortality due to TBI [6].

Despite this, a major criticism of the Brain Trauma Foundation CPGs is that they may not be appropriate for use in all locales due to differences in available resources. Subsequently, a number of newer CPGs have been developed in many different countries and by various practitioner groups based on data and capabilities specific to their respective practice environments [14–16]. Applying these CPGs in LMICs with limited resources is challenging. Additionally, these CPGs vary in quality and content, span across multiple disciplines, and are published in disparate literature bases, making effective utilization challenging. Previous studies evaluating quality of existing TBI CPGs, for instance, have focused on subsets of TBI severity such as mild TBI only [14, 16], or review only a limited number of CPGs [17].

We aimed to assess and summarize the quality of all currently available international acute TBI CPGs by conducting a systematic review using the Appraisal of Guidelines for Research and Evaluation (AGREE) II instrument [17]. We also compared the quality of CPGs created in high resource countries with those from low and middle income. We expect that these results will inform the development of a standard CPG for the acute management of TBI that can be implemented specifically for limited resource settings.

## Materials and Methods

### Protocol and Registration

This systematic review is reported in accordance with the Preferred Reporting Items for Systematic Review and Meta-Analyses (PRISMA) Statement [18], and is registered in the PROSPERO database (International Prospective Register of Systematic Reviews) under the number CRD42013006008.

### Eligibility Criteria

Articles mentioning clinical practice guidelines or recommendations for traumatic brain injury, which met the following inclusion criteria were considered: acute management of TBI (less than 24 hours), any level of traumatic brain injury, high and low income countries and publication in English. No CPG population age restrictions were added as we chose to evaluate both pediatric and adult CPGs. For multiple versions of CPGs, only the newest CPGs were included in the analysis and the older versions were excluded.

### Information Sources

We employed an extensive search strategy from April 2013 to December 2015 to identify guidelines from the following electronic databases: MEDLINE, EMBASE and the Duke University Medical Center Guidelines located in the Duke University Medical Center Library and Archives [19]. The Duke University Medical Center Guidelines is an electronic repository curated by Duke University which gathers several national sources indexing CPGs, including the following: National Guideline Clearinghouse, Center for Disease Control Guidelines, Cochrane Library, AHRQ Evidence reports, American College of Emergency Medicine CPGs, American Academy of Child & Adolescent Psychiatry, American Psychiatric Association Guidelines, Canadian Medical Association, Health Services/Technology Assessment Text Collection, Infectious Disease Society of America, Practice guidelines, Joint National Committee, National Comprehensive Cancer Network Guidelines, National Institute for for Health and Clinical Excellence guidelines, NIH Consensus statements archive, US Preventative Services Task Force, Veterans Affairs Clinical Practice Guidelines. In addition, we manually searched the references and performed a citation analysis of the included studies using Google Scholar to include any potential CPG document that was not included in the initial steps.

### Literature Search

The initial search comprised of the MeSH terms "Brain Injuries", "Guideline [Publication Type]”, “Practice Guideline [Publication Type]”, “Practice Guidelines as Topic”, and their respectives entry terms. Appendix 1 presents the search strategy used in the PubMed database. We did not use limits for languages or date when searching the databases, but added to the final list only documents in English.

### Study Selection

Titles and abstract of the retrieved articles were independently evaluated by two reviewers (A.P. and J.A.). Abstracts that did not provide enough information regarding the eligibility criteria were retrieved for full-text evaluation. Reviewers (A.P. and J.A.) independently evaluated full-text articles and determined study eligibility. Disagreements were solved by consensus and if disagreement persisted, a third reviewer’s opinion was sought (C.S). For CPGs with more than one version, we reported only the most recent versions given their large overlap and anticipated improvement in quality.

### Quality of Clinical Practice Guidelines

Five appraisers independently assessed each eligible and selected guideline for quality in accordance with the AGREE II instrument [17, 20]. AGREE II is an instrument designed to assess quality of clinical practice guidelines. It consists of 23 items divided into six domains: 1) scope and purpose, 2) stakeholder involvement, 3) rigor of development, 4) clarity of presentation, 5) applicability, and 6) editorial independence. Domain one, scope and purpose, evaluates specificity of the overall objective, clinical questions and the patient population described in the CPG. Domain two measures stakeholder involvement, inclusion of relevant professional groups, patients’ views and preferences, and target users. Domain three evaluates the guideline’s systematic methods, stating criteria for selecting evidence and explicit links between evidence and recommendations, strengths and limitations of the evidence, consideration of risks and benefits, external review prior to publication, and procedures for updating the guideline. The fourth domain, clarity of presentation, evaluates for clarity, lack of ambiguity, determines if different management options are presented, and assesses whether key recommendations are easily identifiable. Domain five assesses if the CPGs address recommendations for clinical application, barriers to application, cost or resource implications, and monitoring criteria. Finally, domain six evaluates for editorial independence from funding bodies and addressing of potential conflicts of interest.

Each item was scored on a seven-point scale (one = strongly disagree and seven = strongly agree). Scores for each domain were calculated by using the sum of all items within a domain and scaling the score as a percentage of the maximum possible score using the following formula:

$$\frac{Obtained\;Score\;-\;Minimum\;Possible\;Score}{Maximum\;Possible\;Score-\;Minimum\;Possible\;Score}$$

The results from each guideline were summarized in a heatmap visualization with values for each domain. All 23 items of the AGREE-II instrument were assessed with results reported in percentage form for each of the six domains. A value of 100% indicated a domain in which all items were scored with seven points (strongly agree). A value of 0% corresponded to a domain in which all items were scored with one point (strongly disagree).

Each reviewer received a user’s manual of the AGREE II instrument, containing its instructions. The six AGREE domains were reported independently for each included CPG. Additionally, an independent global assessment was conducted from the six domains and reported along with the appraiser's recommendation. Recommendations were measured in a three-option scale (“Yes”, “Yes with modifications”, and “No”), with a qualitative comment about the CPG [17]. For each domain and for the global assessment, we report average values with respective standard deviations and range (minimum and maximum values).

### Data Extraction

Two reviewers (A.P. and J.A.) independently conducted the data extraction and disagreements were resolved by a third reviewer (C.S.). Besides the AGREE assessment previously described, the following general characteristics of the studies were collected: year of publication, location where the guideline creation took place, the organization that created the guidelines, the main focus of the guideline, the patient population of the guideline, and the severity of TBI the guideline was addressing. Additionally, the countries of origin for each guideline were classified into high-, middle-, and low-income based on World Bank definitions [21]. The senior researcher (C.S.) moderated all discrepancies or doubts during the rating process.

### Data Analysis

Data analysis was performed descriptively and with graphical representation. Overall assessments for each domain were calculated following the methods already described [17, 20]. Consistency of evaluations of the AGREE II domain and for the overall assessment was calculated with an intra-class correlation coefficient (ICC). Graphical solutions were carried out with R software for statistical language [22].

## Results

### Study Selection

**Fig 1:**
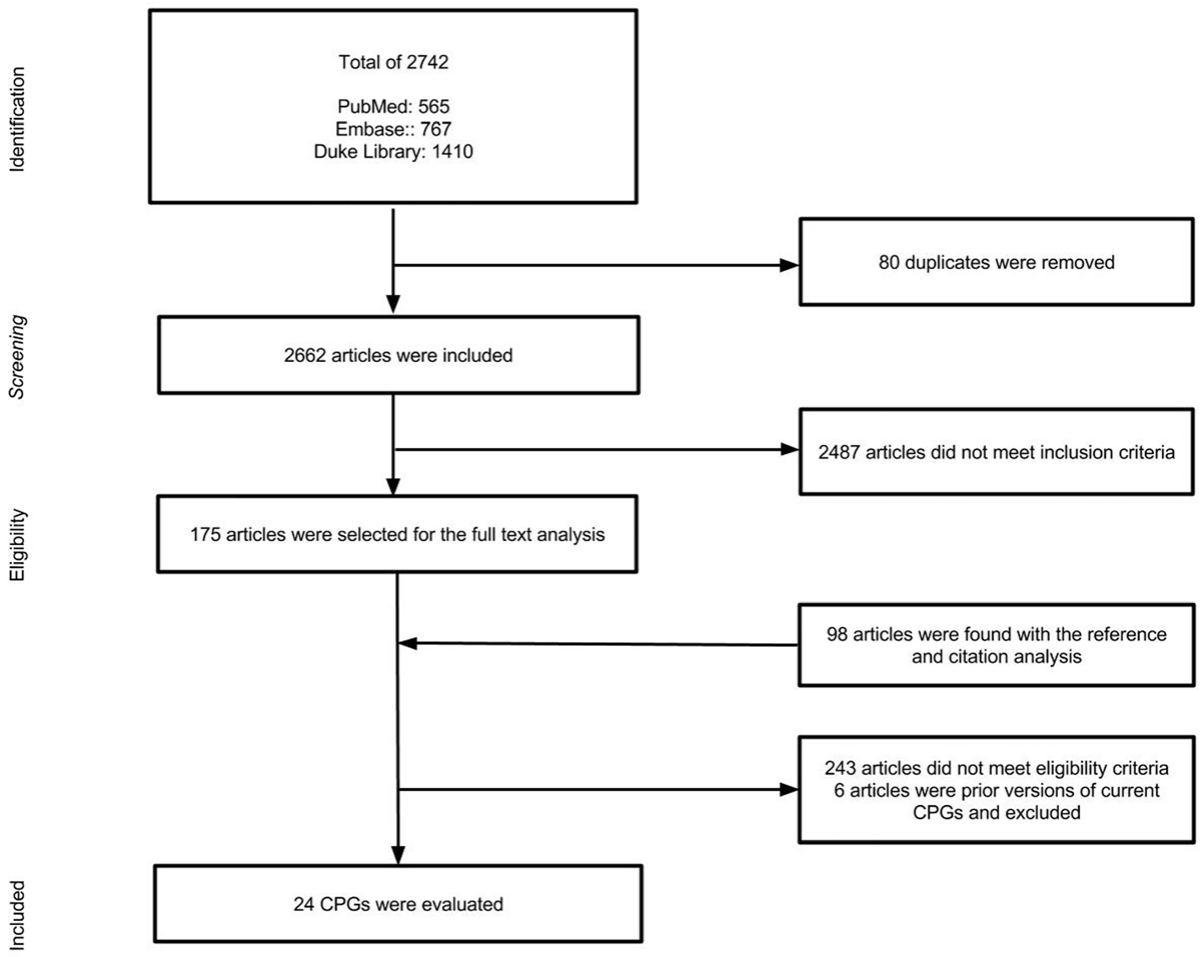
Study Flow Diagram.

The initial search strategy identified 2,742 titles and abstracts 80 of which were removed for duplicates. From these, 2487 were excluded after reviewing abstracts. A reference and citation analysis was performed on the remaining 175 articles yielding an additional 98 abstracts. Full text analysis was then performed on a total of 273 articles of which only 24 [23–46] met inclusion criteria (Fig 1).

### CPG Characteristics

Overall, there were 24 guidelines that were included in this analysis (Table 1) representing 19 different organizations and spanning several countries on four continents. Of these 24 CPG's, 23 [23–45] were developed in high-income countries and only one [46] from a upper middle-income country (Brazil). The CPGs evaluated covered the full scope of adult and pediatric populations with four covering pediatric patients [23, 24, 27, 29], eight for adults patients [25, 28, 33, 38, 40, 41, 43, 44], and seven covering both populations [26, 30, 34, 36, 37, 39, 42]. Five did not specify their population group [31, 32, 35, 45, 46]. Regarding the severity of TBI, one third of the CPGs were developed for minor or mild TBI [23, 24, 25, 29, 31, 32, 34, 33], another third covered severe TBI [26, 27, 28, 33, 35, 36, 37, 44, 45] and the rest were developed for all levels of TBI severity [30, 37, 38, 39, 40, 41, 42]. The majority (19) of CPGs focused on the early management of TBI [23, 24, 25, 27, 29, 30, 31, 32, 34, 38, 39, 40, 41, 42, 43, 44, 46], with two focusing specifically on prehospital care [27, 36], another two on both early management and ICU care [28, 45], one covered prehospital, early management, and rehabilitation [39], and one covered the entire breadth of management of severe TBI [36]. Of the 24 assessed CPGs, roughly half (11) were developed by professional organizations [23, 24, 25, 30, 31, 32, 35, 36, 37, 43, 44], four were developed by non-profit organizations [26, 27, 39, 42], three by international committees [41, 33, 34], another three by national institutes or government organizations [37, 38, 40], one from an academic organization [29], one did not specified the type of organization [45], and the remainder of the CPGs were developed by mixture of different organizations: a non-profit professional organization [28] and a professional organization with an academic organization [46].

**Table 1:** Studies Characteristics.

Guideline Title	Origin	Year of publication	CPG Name	Institution/ Guideline Development group	Type of Institution/ Guideline Development group	Focus of the guideline	Patient population	Severity of brain injury	Country income
Evaluation and management of children younger than two years old with apparently minor head trauma: proposed guidelines. Pediatrics. [23]	USA	2001	AAP, 2001	American Academy of Pediatrics (AAP)	Professional organization	Early management	Pediatrics	Minor	HIC
Committee on Quality Improvement, American Academy of Pediatrics; Commission on Clinical Policies and Research, American Academy of Family Physicians. The management of minor closed head injury in children. Pediatrics. [24]	USA	1999	AAP/AAFP, 1999	American Academy of Pediatrics/American Academy of Family Physicians (AAP/AAFP)	Professional organization	Early management	Pediatrics	Minor	HIC
ACEP Clinical Policy: Neuroimaging and Decisionmaking in Adult Mild Traumatic Brain Injury in the Acute Setting*. [25]	USA	2009	ACEP, 2009	American College of Emergency Physicians	Professional organization	Early management and Imaging diagnosis	Adults	Mild	HIC
Guidelines for prehospital management of traumatic brain injury 2nd edition*. [26]	USA	2007	BTF, 2007	Brain Trauma Foundation (BTF)	Non-profit	Prehospital management	Adults and pediatrics	Severe	HIC
Guidelines for the acute medical management of severe traumatic brain injury in infants, children, and adolescents. [27]	UK	2012	BTF, 2012	Brain Trauma Foundation (BTF)	Non-profit	Early management	Pediatrics	Severe	HIC
Guidelines for the management of severe traumatic brain injury. 3rd edition*. [28]	USA	2007	BTF/AANS, 2007	Brain Trauma Foundation/ American Association of Neurological Surgeons	Non-profit/professional organization	Early management, and ICU care	Adults	Severe	HIC
Mild traumatic brain injury in children: practice guidelines for emergency department and hospitalized patients The Trauma Program, The Children’s Hospital of Philadelphia, University of Pennsylvania School of Medicine. [29]	USA	2003	CHOP, 2003	Trauma Program, The Children's Hospital of Philadelphia, University of Pennsylvania School of Medicine.	Academic institution	Early management	Pediatrics	Mild	HIC
Development of a provincial guideline for the acute assessment and management of adult and pediatric patients with head injuries. [30]	Canada (Nova Scotia)	2007	CMA, 2007	Canadian Medical Association	Professional organization	Early management	Adults and pediatrics	All levels	HIC
Mild Traumatic Brain Injury, Evaluation and Management of (EAST Trauma Guidelines) 2012*. [31]	USA	2012	EAST, 2012	EAST	Professional organization	Early management	Did not specify	Mild	HIC
Practice Management Guidelines for the Management of Mild Traumatic Brain Injury: EAST Practice Management Guidelines Work Group. [32]	USA	2002	EAST, 2002	EAST	Professional organization	Early management	Did not specify	Mild	HIC
EBIC-guidelines for management of severe head injury in adults. [33]	Europe	1997	EBIC, 1997	European Brain Consortium	International committee	Prehospital management, early management, and ICU care	Adults	Severe	HIC
EFNS guideline on mild traumatic brain injury: report of an EFNS task force 2011. [34]	Europe	2011	EFNS, 2011	European Federation of Neurological Societies	International committee	Early management and Imaging diagnosis	Adults and pediatrics	Mild	HIC
Guidelines for the pre-hospital care of patients with severe head injuries. Piek J on behalf of the Working Group for Neurosurgical Intensive Care of the ESICM. [35]	Europe	1998	ESICM, 1998	Working Group for Neurosurgical Intensive Care of the European Society of Intensive Care Medicine	Professional organization	Prehospital management	Did not specify	Severe	HIC
Guidelines for the Management of Severe Head Injury, 2nd edition. Japan Society of Neurotraumatology*. [36]	Japan	2006	JSN, 2006	Japan Society of Neurotraumatology	Professional organization	Management	Adults and pediatrics	Severe	HIC
National Institute for Health and Clinical Excellence. Head injury. Triage, assessment, investigation and early management of head injury in infants, children, and adult: 2007. [37]	UK	2007	NIHCE, 2007	National Institute for Health and Clinical Excellence (NIHCE)	National Institute	Early management	Adults and pediatrics	All levels	HIC
Adult Trauma Clinical Practice Guidelines, Initial Management of Closed Head Injury in Adults. NSW Institute of Trauma and Injury Management. [38]	New South Wales, Australia	2011	NSW MoH, 2011	NSW Ministry of Health	Government organization	Early management of head injury patients	Adults	All levels	HIC
New Zealand Guidelines Group: Traumatic Brain Injury: Diagnosis, Acute Management and Rehabilitation. [39]	Wellington, New Zealand	2006	NZ, 2006	New Zealand Guidelines Group	Non-profit	Acute management, Prehospital management, and rehabilitation process.	Adults and pediatrics	All levels	HIC
Treatment of minor and severe traumatic brain injury. National reference guidelines. [40]	Italy	2008	RHSA, 2008	Regional Healthcare Service Agency (requested by Ministry of Health)	Government organization	Early management	Adults	All levels	HIC
Scandinavian guidelines for initial management of minimal, mild and moderate head injuries in adults: an evidence and consensus-based update. [41]	Scandinavia (Norway, Sweden and Finland)	2013	SCN, 2013	Scandinavian Neurotrauma Committee	International committee	Early management	Adults	All levels	HIC
Early Management of Patients with a Head Injury. A National Clinical Guideline. [42]	Edinburgh, Scotland	2009	SIGN, 2009	Scottish Intercollegiate Guidelines Network	Non-profit	Early management	Adults and pediatrics	All levels	HIC
The Study Group on Head Injury of the Italian Society for Neurosurgery: Guidelines for minor head injured patients' management in adult age. [43]	Italy	1996	SINch, 1996	Italian Society for Neurosurgery	Professional organization	Early management	Adults	Mild	HIC
Guidelines for the treatment of adults with severe head trauma (part I). Initial assessment; evaluation and pre-hospital treatment; current criteria for hospital admission; systemic and cerebral monitoring. [44]	Italy	2000	SINch/SIAARTI, 2000	Italian Society of Neurosurgery (SINch) and Italian Society of Anesthesiology and Intensive Care (SIAARTI)	Professional organization	Early management of severe TBI	Adults	Severe	HIC
Clinical practice guidelines in severe traumatic brain injury in Taiwan. [45]	Taiwan	2009	Taiwan, 2009	Taiwan	Did not specify	Early management, and ICU care	Did not specify	Severe	HIC
Guidelines for Neurosurgical Trauma in Brazil. [46]	Brazil	2001	USP/BSN, 2001	University of São Paulo Medical School/Brazilian Society of Neurosurgery (USP/ BSN)	Academic institution/professional group	Early management	Did not specify	All levels	UMIC

* Clinical Practice Guidelines have older versions, only most recent version of this Clincal Practice Guidelines was included

### CPG Quality Assessment (AGREE)

**Fig 2:**
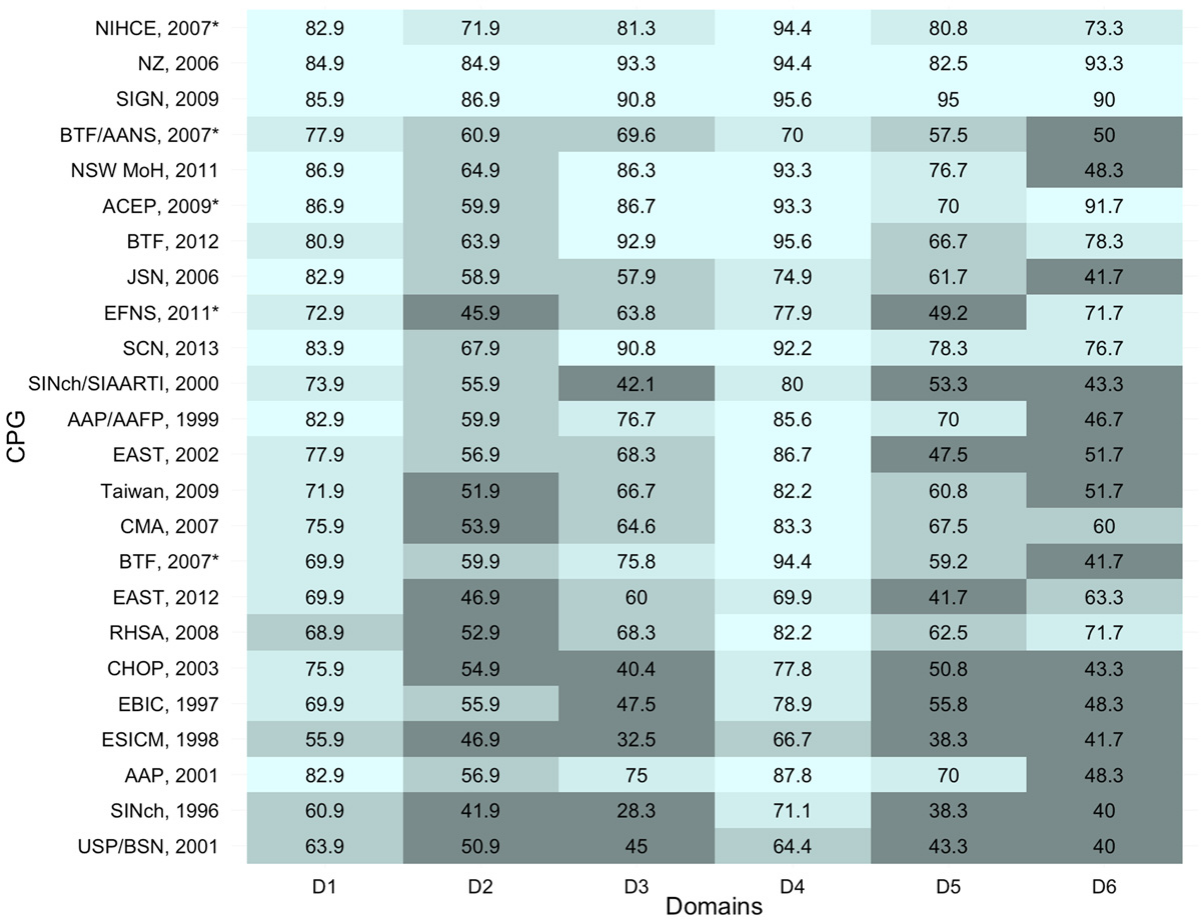
AGREE Scoring by domain for each Clinical Practice Guidelines. (CPG = Clinical Pratice Guideline; D1 = Domain one, scope and porpouse; D2 = Domain 2, stakeholder involvement; D3 = Domain three, rigor of development; D4 = Domain four, clarity of presentation; D5 = Domain five, applicability; D6 = Domain six, editorial Independence, * = Indicates newest version of a CPG for which multiple versions exist).

All AGREE II assessments summaries (average per domain and standard deviation) are described per domain and with the overall assessment. Domain specific results are summarized in Fig 2. Overall assessment recommendations and comments are displayed in Table 2. Inter-rater reliability for each domain is expressed in Table 3.

**Table 2:** Clinical practice guideline overall assessment, recommendation evaluation and comments themes.

CPGs	Overall Assessment					% of CPG recommendation for use			Summary of the appraisers' comments
	A1	A2	A3	A4	A5	Yes	Mod.	No	
AAP, 2001	6	7	5	4	6	100%	0%	0%	Well written and comprehensive text with good levels of evidence. Average methods and stakeholder involvement. Merits in use despite population age. Presence of good facilitators for applicability.
AAP/AAFP, 1999	6	5	6	5	5	60%	20%	20%	Well writtten and comprehensive text with good levels of evidence. Average methods and applicabilty.
ACEP, 2009	6	7	6	6	6	100%	0%	0%	Well written and comprehensive text with good levels of evidence. Average guideline's updating system and stakeholder involvement.
BTF, 2007	5	6	3	5	5	60%	20%	20%	Comprehensive text with average levels of evidence. Limited by demography (varying resources in Italy). Good applicabilty. The text has good informations for prehospital care but have average informations about the monitoring criteria.
BTF, 2012	6	7	6	6	6	100%	0%	0%	Comprehensive text with good levels of evidence and population focus (pediatrics). Average applicabilty and stakeholder involvement.
BTF/AANS, 2007	6	7	6	7	7	100%	0%	0%	Well written and comprehensive text with good levels of evidence. Good methods but average applicability. No conflicts of interest but resource-limited enviroment.
CHOP, 2003	4	5	5	3	6	20%	60%	20%	Bad levels of evidence with unclear methods. Average applicability and stakeholder involviment.
CMA, 2007	4	6	6	4	5	60%	20%	20%	Comprehensive text with good levels of evidence and unclear methods. Average guideline's uptodating system and monitoring criteria. The scope is limited though acknowledged and stakeholder involvement.
EAST, 2002	5	6	6	6	4	20%	60%	20%	Well written and comprehensive text. Unclear levels of evidence. Poor discussion on implementation and recommendations. Bad methods, applicability and stakeholder involviment.
EAST, 2012	5	6	5	3	5	60%	40%	0%	Comprehensive text with good levels of evidence but unclear selection criteria for the evidence. No external review. Good methods and scope.
EBIC, 1997	3	6	5	1	5	60%	0%	40%	Bad levels of evidence, population remains unclear. Scope is limited by region. Bad applicabilty and methods.
EFNS, 2001	5	6	7	6	5	100%	0%	0%	Comprehensive text with good levels of evidence. Presence of good facilitators for applicability. Good scope and methods.
ESICM, 1998	4	3	7	3	3	20%	20%	60%	Comprehensive text with average levels of evidence. Bad methods and applicability. Low content paper.
JSN, 2006	5	7	5	7	6	60%	40%	0%	Well written and comprehensive text with average evidence. Good methods. Scope is limited the due age of the population.
NIHCE, 2007	7	7	7	7	7	100%	0%	0%	Well written text. Good levels of evidence, scope and methods.
NSW MoH, 2011	7	6	7	5	7	100%	0%	0%	Well written and comprehensive text with good levels of evidence. Good applicability. Average stakeholder involvement and editorial independence. Unclear guideline updating system.
NZ, 2006	6	7	7	7	7	100%	0%	0%	Well written and comprehensive text with good levels of evidence. High lenght.
RHSA, 2008	3	6	6	6	3	60%	40%	0%	Unclear understanding text with unclear levels of evidence. Average guideline updating system. Good scope. Bad stakeholder involvement.
SCN, 2013	6	7	5	6	5	80%	20%	0%	Well written and comprehensive text with unclear levels of evidence (do not specify the class of evidence). Average stakeholder involvement.
SIGN, 2009	6	7	7	7	7	100%	0%	0%	Well written and comprehensive text with good evidence based. Good applicability. No competing interests by contributors.
SINch, 1996	2	3	7	2	3	0%	0%	100%	Bad levels of evidence and is outdated.
SINch/SIAARTI, 2000	4	5	7	3	6	20%	60%	20%	Well writen and comprehensive text
Taiwan, 2009	5	6	7	5	4	60%	40%	0%	Comprehensive text with good levels of eviedence. The paper needs more informations on the monitoring criteria. High lengh text.
USP/BSN, 2001	3	4	7	3	5	20%	20%	60%	Well written and comprehensive text. Unclear methods. Bad applicability.

(CPGs = Clinical Pratice Guidelines; A1, A2, A3, A4, A5 = appraisers 1 to 5 respectively)

**Table 3:** Inter-rater reliability for each AGREE quality domain.

Domains	ICC	95% CI
Scope and purpose	0.65*	(0.37;0.83)
Stakeholder involvement	0.78*	(0.60;0.89)
Rigor of development	0.96*	(0.93;0.98)
Clarity and presentation	0.43*	(-0.04;0.72)
Applicability	0.64*	(0.34;0.82)
Editorial independence	0.73*	(0.51;0.87)
Overall assessment	0.79*	(0.62;0.90)

* significant to p<0.05. ICC = intra-class correlation coefficient. 95% CI = 95% confidence interval.

#### Domain One—Scope and Purpose

The lowest score was 55.9 which was from Guidelines for the Pre-hospital Care of Patients with Severe Head Injuries (ESICM, 1998). The highest score was 86.9, from both the Adult Trauma Clinical Practice Guidelines, Initial Management of Closed Head Injury in Adults (NSW MoH, 2011) and the ACEP Clinical Policy: Neuroimaging and Decisionmaking in Adult Mild Traumatic Brain Injury in the Acute Setting (ACEP, 2009).

#### Domain Two—Stakeholder Involvement

The highest score of 86.9 was from the Early Management of Patients with a Head Injury; A National Clinical Guideline. (SIGN, 2009) The lowest score of 41.9 was from The Study Group on Head Injury of the Italian Society for Neurosurgery: Guidelines for minor head injured patients' management in adult age (SINch, 1996).

#### Domain Three—Rigor of Development

The lowest score was 28.3 from The Study Group on Head Injury of the Italian Society for Neurosurgery: Guidelines for minor head injured patients' management in adult age (SINch, 1996). The highest score of 93.3 was from the New Zealand Guidelines Group: Traumatic Brain Injury: Diagnosis, Acute Management and Rehabilitation (NZ, 2006).

#### Domain Four—Clarity of Presentation

The lowest score was 64.4 from the Guidelines for Neurosurgical Trauma in Brazil (USP/BSN, 2001) and the highest value was 95.6 achieved by two guidelines: Early Management of Patients with a Head Injury: A National Clinical Guideline (SIGN, 2009) and the Guidelines for the Acute Medical Management of Severe Traumatic Brain Injury in Infants, Children, and Adolescents (BTF, 2012).

#### Domain Five–Application

Two guidelines shared the lowest score (38.3): the Study Group on Head Injury of the Italian Society for Neurosurgery: Guidelines for Minor Head Injured Patients’ Management in Adult Age (SINch, 1996) and Guidelines for the Pre-hospital care of Patients with Severe Head Injuries (ESICM, 1998). The highest score of 95.0 belonged to the following guideline: Early Management of Patients with a Head Injury. A National Clinical Guideline (SIGN, 2009).

#### Domain Six—Editorial Independence

The lowest score of 40 points came from both the Study Group on Head Injury of the Italian Society for Neurosurgery: Guidelines for minor head injured patients' management in adult age (SINch, 1996) and the Guidelines for Neurosurgical Trauma in Brazil (USP/BSN, 2001). The highest score was 93.3, from the New Zealand Guidelines Group: Traumatic Brain Injury: Diagnosis, Acute Management and Rehabilitation (NZ, 2006).

#### Overall Assessment

The 2007 CPG from the National Institute for Health and Clinical Excellence (NIHCE, 2007) had the best overall assessment with maximum score from all appraisers, followed by the New Zealand Guidelines Group (NZ, 2006) and the National Clinical Guideline (SIGN, 2009) both with only one appraiser scoring six points each. The worst ovserall assessment was for the CPG from The Study Group on Head Injury of the Italian Society for Neurosurgery (SINch, 1996). Most CPGs would be recommend by the appraisers (EFNS, 2001; AAP, 2001; NZ, 2006; BTF/AANS, 2007; NIHCE, 2007; ACEP, 2009; SIGN, 2009; NSW MoH, 2011). Three CPGs had 60% of appraisers recommending with modifications (SINch/SIAARTI, 2000; EAST, 2002; CHOP, 2003). The guidelines from the Study Group on Head Injury of the Italian Society for Neurosurgery (SINch, 1996) was the only CPG that 100% of reviewers reported they would not recommend for use. Comments on the strengths and weaknesses of each CPG are summarized in Table 2.

#### Appraisers Consistency

Overall reliability was very good, with three of the quality domains (stakeholder involvement, rigor of development and editorial independence) and the overall assessment with values above 0.70, and two (scope and purpose and applicability) values around 0.65. The domain “clarity and presentation” had the lowest reliability (0.43) (Table 3).

## Discussion

This systematic review is the first to synthesize and collate all published clinical practice guidelines for all types of traumatic brain injury into a single large-scale quality review. Our study included 24 CPGs; across all TBI CPGs, the highest mean scores were achieved in clarity and presentation, scope and purpose and rigour of development, while the main weaknesses across CPGs were stakeholder involvement, applicability and editorial independence. The National Institute for Health and Clinical Excellence (NIHCE, 2007) guidelines, the New Zealand Guidelines Group (NZ, 2006), and the National Clinical Guideline (SIGN, 2009) were the three CPGs with best results. The majority of CPGs evaluated in this study were developed by high income countries (HICs), and are therefore minimally applicable in resource limited settings.

### CPGs strengths and weaknesses

Overall, the strong scores in the clarity and presentation, scope and purpose, and rigor of development domains have been reported in other systematic reviews evaluating TBI CPGs [14, 17, 47, 48]. This is likely attributed to the scientific rigor of developing a CPG, which typically involves a highly methodical approach [49]. In general, the guidelines that were more recently developed or updated, and those that had undergone numerous updates, most consistently demonstrated the highest quality by AGREE II scores.

Our analysis indicates an overall improvement in the above domains in the most current CPGs, consistent with other studies [14–16, 50]. In a 2011 systematic review of CPGs for managing mild TBI by Tavender et al, the NSW 2006 CPGs fared worse in all domains with the exception of domain scope and purpose when compared to our values for the 2011 version of the guidelines [14]. Similarly, another systematic review evaluated an older 2003 version of NIHCE determining an overall assessment score of 66.9 compared to a score of 100.0 on the 2007 version in our study [17]. It is noteworthy to mention that more recent CPGs have also the advantage of newer and more rigorous evidence-based medicine in addition to the availability of designing guidelines around the AGREE or AGREE II format. Nevertheless, a frequently criticized area in our results, within the rigor of development domain, was the lack of procedures for updating the guidelines for quality improvement (QI). Given the trend toward improved CPG quality with newer revisions, development of a systematic QI procedure may help to ensure quality of future CPGs without the resources required for a full new edition every few years.

Older reviews have demonstrated limited Stakeholder involvement in CPG development, a trend that persists in our current review of CPGs [14, 15]. While there has been progressive improvement in CPG development, the domains of stakeholder involvement, applicability, and editorial independence remain weak, specifically when it comes to piloting interventions, addressing potential costs and barriers to implementation, and auditing for quality improvement. Recent literature suggests that successful implementation of CPGs reduces mortality and morbidity [6, 51–54], however applicability of guidelines to a given locale based on factors such as availability and cost of resources, provider skills, and population needs and values, are critically important for successful implementation of CPGs in a manner that will improve patient care. Consideration of stakeholder involvement and applicability are imperative considering these domains are intrinsically associated with CPG implementation and translation to other settings such as LMICs.

It has been suggested that adaptation of existing CPGs to local situations may be a more valid and cost-effective means of achieving high-quality CPGs worldwide [55]. However, recent attempts have revealed that this adaptation process remains complex and challenging, requiring careful planning and implementation to avoid additional costly resource utilization [56]. In particular, when the CPG recommendations require resources not present in a given locale, alternatives to the suggested “optimal” recommendations are required. Given our findings that most CPGs lacked information on addressing costs and barriers to implementation, such as in resource-poor settings, this demonstrates an important void in existing CPGs. Current attempts at adapting existing CPGs may require local practitioners and CPG developers to return to the primary literature to develop location-specific alternatives that are more practicable in their region or medical center. In addition, further research may be required to develop these potential cost-reducing alternatives and demonstrate efficacy.

### CPGs and relevance to LMICs

As identified in this systematic review most CPGs have been developed in HICs, which makes them of questionable relevance to LMICs, especially for those populations with different cost-benefit parameters for medical care. Many hospitals in LMIC, particularly in more rural areas, lack basic intensive or critical care capabilities, specialized staff, or even necessary diagnostic imaging, factors upon which the majority of the highest scoring guidelines rely upon for implementation [57].

LMICs have potentially greater challenges and barriers to implementation than HICs based on these factors, which need to be addressed to enable a CPG to be useful and beneficial. The few CPGs with high applicability scores were developed by and tailored for HICs [34, 35], which makes them unlikely to be applicable for use in limited resourced settings and LMIC countries in their current forms.

### Individual CPGs quality assessment and previous research

Comparing our results with other systematic reviews found many similarities. The EAST Evaluation and Management of the Mild Traumatic Brain Injury CPG (EAST, 2012) had similar evaluation of the best and worst domains. Our assessment of the Guidelines for the Acute Medical Management of Severe Traumatic Brain Injury in Infants, Children, and Adolescents (BTF, 2012) was similar to that of Grimmer et al., only differing in the applicability domain, which they assigned a fairly low score of 26.4, compared to our value of 66.7. However, the overall assessment was similar [24]. We found similar evaluations for domains, overall assessment and recommendations for the CPG from the National Institute for Health and Clinical Excellence (NIHCE, 2007) [14, 17, 47, 48, 58] and the National Clinical Guideline (SIGN, 2009) [14, 58] in other studies.

We did find some differences in the domains of applicability and editorial independence in the systematic review mild TBI CPGs by Tavender et al. For the ACEP Clinical Policy, we assessed scores of 70 and 91.7 in the fields of applicability and editorial independence, respectively, compared to their findings of 17 and 50 [14]. The same study, in addition to a 2011 evaluation by Berrigan et al. found respective scores of 36 and 50 in the applicability domain for the New Zealand Guidelines Group, whereas our score was much higher at 82.5 [14, 47]. Given the limited number of systematic reviews on this topic, we were not able to compare all CPGs included in this study.

#### Limitations

The main limitations of our study are the subjectiveness of the AGREE II tool and the potential bias of the reviewers performing the assessment. The AGREE II tool is a 23 question tool established to evaluate CPG quality. While it is a subjective tool, it is the current gold standard; the AGREE II guidelines suggest using at least two and preferably four appraisers with content specific knowledge [17]. We utilized five appraisers who all had content specific knowledge; four were emergency medicine physicians with experience in research and evidence-based methods and one was a masters level student with extensive research and content topic experience. Given that the AGREE II assessment requires evaluation of the CPGs based on the descriptions available in the published manuscripts, there is a small chance that inaccurate assessments would be due to poor descriptions in the manuscript. However, comparison of our AGREE II ratings to those of other researchers found similar scores [14].

Expanding the reviewers to include other relevant specialties such as neurosurgery, critical care, or neurology would have provided additional input on specialty-specific recommendations. Additionally, due to language limitations, we were only able to review CPGs in English. The language limitation did not provide a significant barrier to most CPGs, given that many of the articles written in different languages were also available in English, or failed to meet inclusion criteria. However, given that many LMICs are non-English speaking, they may have developed CPGs in other languages that we were unable to evaluate and subsequently missed. Finally, we did not search some clinical databases, like the TRIP and GIN repository. However, due to the several diverse repositories included through the Duke library search, it is unlikely that we missed published CPGs.

#### Conclusions

Our review identifies two specific areas for improvement in clinical practice guidelines addressing the acute management of TBI: (a) the domains of stakeholder involvement, applicability, and editorial independence remain weak and insufficiently described specifically when it comes to piloting interventions, addressing potential costs and implementation barriers, and auditing for quality improvement; (b) CPGs created specifically for use in low income settings are non-existent. Most of these CPGs were developed by high-income countries with only one CPG from an upper middle income country, which was found to have a poorer quality across all domains. This will limit the applicability and implementation capacity of CPGs for limited resourced settings.

## Supporting Information

S1 Appendix Search strategy for PubMed. (DOCX)

S2 Appendix PRISMA 2009 Checklist. (DOCX)
