# Supplementary material for: Quality of the Development of Traumatic Brain Injury Clinical Practice Guidelines: A Systematic Review
Source: PLoS One. 2016 Sep 1;11(9):e0161554. doi: 10.1371/journal.pone.0161554 (PMC5008729; doi:10.1371/journal.pone.0161554)
Supplement: S1 Appendix — (DOCX) [file pone.0161554.s001.docx]

### Pubmed - 378 results

| Search | Add to builder | Query | Items found |
| --- | --- | --- | --- |
| [#3http://www.ncbi.nlm.nih.gov/pubmed](http://www.ncbi.nlm.nih.gov/pubmed) | [Addhttp://www.ncbi.nlm.nih.gov/pubmed](http://www.ncbi.nlm.nih.gov/pubmed) | Search **#1 AND #2** | [378http://www.ncbi.nlm.nih.gov/pubmed/?cmd=HistorySearch&querykey=13](http://www.ncbi.nlm.nih.gov/pubmed/?cmd=HistorySearch&querykey=13) |
| [#2http://www.ncbi.nlm.nih.gov/pubmed](http://www.ncbi.nlm.nih.gov/pubmed) | [Addhttp://www.ncbi.nlm.nih.gov/pubmed](http://www.ncbi.nlm.nih.gov/pubmed) | Search **“Brain Injuries[Mesh Term] OR “Brain Injuries[All Terms] OR “Brain Injury”[All Terms] OR “Injury, Brain”[All Terms] OR “Injuries, Brain”[All Terms] OR “Brain Injuries, Diffuse”[All Terms] OR “Brain Injury, Diffuse”[All Terms] OR “Diffuse Brain Injuries”[All Terms] OR “Diffuse Brain Injury”[All Terms] OR “Injuries, Diffuse Brain”[All Terms] OR “Injury, Diffuse Brain”[All Terms] OR “Brain Injuries, Focal”[All Terms] OR “Brain Injury, Focal”[All Terms] OR “Focal Brain Injury”[All Terms] OR “Injuries, Focal Brain”[All Terms] OR “Injury, Focal Brain”[All Terms] OR “Focal Brain Injuries”[All Terms] OR “Injuries, Acute Brain”[All Terms] OR “Acute Brain Injury”[All Terms] OR “Brain Injury, Acute”[All Terms] OR “Injury, Acute Brain”[All Terms] OR “Brain Injuries, Acute”[All Terms] OR “Acute Brain Injuries”[All Terms] OR “Brain Lacerations”[All Terms] OR “Brain Laceration”[All Terms] OR “Laceration, Brain”[All Terms] OR “Lacerations, Brain”[All Terms] OR “Cortical Contusion”[All Terms] OR “Contusion, Cortical”[All Terms] OR “Contusions, Cortical”[All Terms] OR “Cortical Contusions”[All Terms] OR “Encephalopathy, Post-Concussive”[All Terms] OR “Encephalopathies, Post-Concussive”[All Terms] OR “Encephalopathy, Post Concussive”[All Terms] OR “Post-Concussive Encephalopathies”[All Terms] OR “Post-Concussive Encephalopathy”[All Terms] OR “Post Concussive Encephalopathy”[All Terms] OR “Encephalopathy, Post-Traumatic”[All Terms] OR “Encephalopathies, Post-Traumatic”[All Terms] OR “Encephalopathy, Post Traumatic”[All Terms] OR “Post-Traumatic Encephalopathies”[All Terms] OR “Post-Traumatic Encephalopathy”[All Terms] OR “Post Traumatic Encephalopathy”[All Terms] OR “Brain Contusion”[All Terms] OR “Brain Contusions”[All Terms] OR “Contusion, Brain”[All Terms] OR “Contusions, Brain”[All Terms] OR “Brain Injuries, Traumatic”[All Terms] OR “Brain Injury, Traumatic”[All Terms] OR “Injuries, Traumatic Brain”[All Terms] OR “Injury, Traumatic Brain”[All Terms] OR “Traumatic Brain Injuries”[All Terms] OR “Injury, Brain, Traumatic”[All Terms] OR “Traumatic Encephalopathy”[All Terms] OR “Trauma, Brain”[All Terms] OR “Brain Trauma”[All Terms] OR “Brain Traumas”[All Terms] OR “Traumas, Brain”[All Terms] OR “Traumatic Brain Injury”[All Terms] OR “Encephalopathy, Traumatic”[All Terms] OR “Encephalopathies, Traumatic”[All Terms] OR “Traumatic Encephalopathies”[All Terms] OR “TBI (Traumatic Brain Injury)”[All Terms] OR “TBIs (Traumatic Brain Injury)”[All Terms]** | [32033http://www.ncbi.nlm.nih.gov/pubmed/?cmd=HistorySearch&querykey=12](http://www.ncbi.nlm.nih.gov/pubmed/?cmd=HistorySearch&querykey=12) |
| [#1http://www.ncbi.nlm.nih.gov/pubmed](http://www.ncbi.nlm.nih.gov/pubmed) | [Addhttp://www.ncbi.nlm.nih.gov/pubmed](http://www.ncbi.nlm.nih.gov/pubmed) | Search **“Guideline [Publication Type]”[Mesh Term] OR “Practice Guideline [Publication Type]”[Mesh Term] OR “Practice Guidelines as Topic”[Mesh Term] OR “Guideline [Publication Type]”[All Terms] OR “Practice Guideline [Publication Type]”[All Terms] OR “Practice Guidelines as Topic”[All Terms] OR “Best Practices”[All Terms] OR “Best Practice”[All Terms] OR “Practice, Best”[All Terms] OR “Practices, Best”[All Terms] OR “Guidelines as Topic”[All Terms]** | [103358http://www.ncbi.nlm.nih.gov/pubmed/?cmd=HistorySearch&querykey=11](http://www.ncbi.nlm.nih.gov/pubmed/?cmd=HistorySearch&querykey=11) |

<http://www.ncbi.nlm.nih.gov/pubmed/?cmd=HistorySearch&querykey=11>

<http://www.ncbi.nlm.nih.gov/pubmed/?cmd=HistorySearch&querykey=11>

### EMBASE - 386 results

'brain injury'/exp AND 'practice guideline'/exp

#1 AND 'practice guideline'/de AND 'traumatic brain injury'/de
